# A retrospective study of psychotropic drug treatments in bipolar disorder at acute and maintenance episodes

**DOI:** 10.3389/fpsyt.2023.1057780

**Published:** 2023-02-07

**Authors:** Pan Jing, Jianjun Su, Chengying Zheng, Xi Mei, Xiaobin Zhang

**Affiliations:** ^1^School of Medicine, Soochow University, Suzhou, China; ^2^Department of Psychiatric, Ningbo Kangning Hospital, Ningbo, China; ^3^Department of Psychiatric, Suzhou Guangji Hospital, Suzhou, China

**Keywords:** psychotropic drugs, bipolar disorder, acute, maintenance, retrospective

## Abstract

**Background:**

Bipolar disorder (BD) is predominantly treated with psychotropic drugs, but BD is a complex medical condition and the contribution of psychotropic drugs is not clear. The objectives of this study are: (1) to present psychotropic drugs used in patients with BD; (2) to access changes of psychotropic drug treatments in acute and maintenance episodes.

**Methods:**

The study retrospectively evaluated the medical records of inpatients in the Ningbo Kangning Hospital from January 2019 to December 2019. The medical history of each subject was collected completely, including sociodemographic (gender, age, marital status, and so on) and clinical characteristics at baseline and within 12 months of admission.

**Results:**

The study ultimately included 204 patients with BD. After 12 months, 73.0% of the patients still took drugs. Mood stabilizers (72–90%) and antipsychotics (77–95%) were still the most important drugs in patients with BD. Antidepressants (34–40%) and benzodiazepines (20–34%) were the other frequently used drug classes. For mood stabilizers, 40–56% of patients were prescribed lithium. For antipsychotic, 54–65% of patients were prescribed quetiapine. Sertraline (6–9%) and fluoxetine (5–9%) were the antidepressant that most frequently prescribed. Lorazepam (10–18%) was the most commonly used benzodiazepine. In psychotropic polypharmacy, the most frequently taken was mood stabilizer plus antipsychotic co-treatment, about 36–44% of all patients. A total of 35–48% of patients treated by two psychotropic drugs and 24–36% received three.

**Conclusion:**

The first 6 months after treatment is very important to medication adherence. Mood stabilizers and antipsychotic remained the primary treatment for BD. Antipsychotic is on the rise in the treatment of BD.

## Introduction

Bipolar disorder (BD) is a common mental illness that affects more than 2% of the world’s population (1). Most affected individuals experience illness-related disability, reduced psychosocial functioning, reduced quality of life, and increased economic costs (2, 3). Mortality studies indicate that BD, like schizophrenia, is associated with a loss of approximately 10–20 potential years of life (4). In addition, people with BD die from suicide more often than people with other mental disorders (5, 6), such as major depressive disorder (MDD). The most important unmet need in BD is accurate and timely diagnosis and prompt implementation of effective treatment (7).

Multiple nationally and internationally authored guidelines for BD have been published in recent years (8–12). The primary modalities of therapy in BD are psychotropic drugs, psychosocial counseling, neurostimulation therapies (e.g., electroconvulsive therapy), and lifestyle modification. Although psychotropic drugs have shown an effect on the neuroendocrine system and other faults (13), psychotropic drugs treatments are the foundation of any treatment plan and have been studied to a greater extent than other treatments. However, the exclusion of BD patients from randomized controlled trials – those most common in clinical practice (e.g., patients with multiple comorbidities and suicidal tendencies) – limits what can be inferred from these study findings to clinical practice (14). It is important to understand the real-world use of psychotropic drugs in patients with BD.

Mood stabilizers and antipsychotics are standard treatment for acute manic episodes (15). For acute BD mania, monotherapy with either mood stabilizers or antipsychotics is recommended, and the combination of a mood stabilizer with an antipsychotic is suggested for cases in which monotherapy is not sufficient (16). Although this polypharmacy treatment is also associated with common comorbidities (17), two or more medications with mood-stabilizing properties are usually part of the treatment regimen for BD (18). Mania is an adverse effect of antidepressant therapy in patients with BD (19). However, antidepressants are commonly used drugs in patients with BD. These are the reasons why antidepressants are commonly used in patients with BD. There are other reasons as well, for example, patients with BD usually develop during episodes of depression or mixed emotions, and depressive symptoms tend to dominate the course of the illness (20, 21). Benzodiazepines are also commonly prescribed to patients with BD. Some studies suggest that some specific anxiety disorders representing the phase of abnormal rhythm within the BD disease spectrum (22).

Few psychotropic drug treatments for BD have shown efficacy as maintenance treatments. In Scotland, the percentage of patients with BD receiving lithium declined between 2009 and 2016 (23). Lamotrigine has shown efficacy as a maintenance therapy for depression (24). Evidence of efficacy as a maintenance treatment for BD exists for quetiapine, but not for risperidone or aripiprazole (25). Maintenance treatments are important to control mood symptoms and to reduce relapse. Therefore, it is important to fully understand the efficacy of psychotropic drugs in maintenance treatment for patients with BD.

The incidence and prevalence of diagnosed BD have increased during the last 20 years (26), not least because of the launch of new treatments. It is not known whether this has affected the prescription pattern. With the expansion of available medications for BD, such as new generation antidepressants and antipsychotics, and the use of psychiatric indications for anticonvulsants, prescribers have many options. Effective treatment for patients with BD and good medication adherence to treatment are important for the prognosis of patients, which can reduce the recurrence rate, improve the quality of life of patients, and reduce the burden on family and society. We selected inpatients, and this study explored drug treatment patterns in BD during acute episodes and maintenance periods of approximately 12 months of psychotropic drug intake. From this study, we want to know the characteristics of psychotropic drug use and medication adherence of patients with BD, so as to provide evidence for psychiatrists in clinical medication.

## Materials and methods

### Data collection

This study was approved by the Ethics Committee of Ningbo Kangning Hospital. The study retrospectively evaluated the medical records of inpatients in Ningbo Kangning Hospital from January 2019 to December 2019. Because this was a retrospective, non-interventional study and all data were collected anonymously, informed consent was not required. All the patients included in the study needed to meet the International Classification of Diseases, Tenth Edition (ICD-10) criteria for BD. The diagnosis of BD was made by a psychiatrist during hospitalization, and all the patients received psychotropic drug treatments. We excluded patients with history of schizophrenia, epilepsy, alcohol, and psychoactive substance dependence, have a history of organic brain disease, serious physical disease or endocrine disease, pregnant or suspected pregnancy patients, and nursing patients. If multiple hospitalizations were found during the time of admission, the record from the most recent hospitalization was selected. If there was a record of hospitalization during the maintenance period, it was ruled out. The study had minimal inclusion criteria to better represent the patient heterogeneity of clinical practice.

The medical history of each subject was collected, including sociodemographic (sex, age, marital status, and so on), and clinical characteristics were assessed at baseline and within 12 months of admission. Subsequent assessment data were collected 3 months after the baseline, at 6 months, at 9 months, and at 12 months.

### Medication classes

The psychotropic drugs mentioned in this study were mood stabilizers (MSs), antipsychotics (APs), antidepressants (ADs), and benzodiazepines (BZDs). Mood stabilizers were defined as valproate, lithium, lamotrigine, oxcarbazepine, or topiramate. Second-generation antipsychotics are also effective mood stabilizers, but they were classified separately in order to better classify the drugs taken and compare them with previous studies. Trazodone and mirtazapine were considered antidepressants, although they were often used to improve sleep. These included benzodiazepines, because they are commonly used in patients with BD. For each patient, the prescribed daily dose (PDD) was defined as the daily dose. The type of medication taken was defined as the daily use of any medication within the type of medication.

Polypharmacy was defined as the use of two or more psychotropic drugs. Polypharmacy might involve the same class of drugs, such as two antidepressants, or different classes of drugs, such as mood stabilizers and antipsychotics. Fifteen types of medication classes were analyzed, including four types of monotherapies, six types with two different classes of drug co-treatments, four types with three different classes of drug co-treatments, and one type with four different classes of drug co-treatment. For all patients, the numbers of drugs used were collected at baseline and at each follow-up assessment. The changes in the drug numbers were observed from the collection of these data.

### Statistical analysis

Data on sociodemographic, clinical characteristics, numbers of different medication classes, their dosage of psychotropic drugs, drug combinations, and number of psychotropic drugs were compared between groups. Continuous variables that did not conform to the normal distribution were reported as median (1st quartile = Q1, 3rd quartile = Q3), and those that conform to the normal distribution were reported as means ± the standard deviation (SD). Categorical variables were reported in numbers (percentages). Chi-square test was used to compare categorical variables. For the most commonly used drugs, only one of the classes of drugs taken by ≥4% or more of the patients was included. If the number of cases was less than 5, then the outcome from Fisher’s exact test was used. Data were analyzed using two-tailed significance estimates. All data was analyzed using SPSS 21.0 software (Statistical Package for Social Sciences, SPSS Inc.) and the statistical significance was set at *P*-value < 0.05.

## Results

The study ultimately included 204 patients with BD, of whom, 108 (52.9%) were diagnosed with bipolar depression, 55 (27.0%) with bipolar mania, 35 (17.2%) with mixed state, and 3 (1.5%) with rapid cycle; 2 (1.0%) were in remission and 1 (0.4%) were not classified. Of the 204 patients, 77 (37.7%) were male and 127 (62.3%) were female, and the age at enrollment was 29.0 (20.0, 44.8) years old; 101 (49.5%) were single, and 83 (40.7%) were married. The median disease course was 48.0 (19.5, 120) months, and the patients reported 21 (13, 32) days of psychiatric hospitalization for BD. The sociodemographic and clinical characteristics of the patients during the maintenance period are shown in Table 1. There were no significant sociodemographic or clinical characteristics among the groups (*P* > 0.05).

**TABLE 1:** Sociodemographic and clinical characteristics of patients with bipolar disorder.

	Acute episode	Maintenance period				χ^2^	*P*
	Baseline (*n* = 204)	3 months (*n* = 173)	6 months (*n* = 153)	9 months (*n* = 147)	12 months (*n* = 149)		
**Sex**							
Male	77 (37.7)	65 (37.6)	56 (36.6)	54 (36.7)	56 (37.6)	0.080	0.999
Female	127 (62.3)	108 (62.4)	97 (63.4)	93 (63.3)	93 (62.4)	0.080	0.999
**Marital status**							
Single	101 (49.5)	82 (47.4)	68 (44.4)	64 (43.5)	63 (42.3)	2.468	0.650
Married/spouse	83 (40.7)	76 (43.9)	70 (45.8)	68 (46.3)	71 (47.7)	2.103	0.717
Divorced	9 (4.4)	6 (3.5)	6 (3.9)	6 (4.1)	6 (4.0)	0.223	0.994
Widowed	8 (3.9)	7 (4.0)	7 (4.6)	7 (4.8)	7 (4.7)	0.250	0.993
Other	3 (1.5)	2 (1.2)	2 (1.3)	2 (1.4)	2 (1.3)	0.413	1.000
**Disease course, months**							
0–12	48 (23.5)	38 (22.0)	30 (19.6)	30 (20.4)	29 (19.5)	1.280	0.865
13–60	73 (35.8)	58 (33.5)	51 (33.3)	46 (31.3)	46 (30.9)	1.229	0.873
61–120	33 (16.2)	29 (16.8)	27 (17.6)	26 (17.7)	27 (18.1)	0.308	0.989
121–240	32 (15.7)	30 (17.3)	27 (17.6)	27 (18.4)	30 (20.1)	1.236	0.872
≥241	18 (8.8)	18 (10.4)	18 (11.8)	18 (12.2)	17 (11.4)	1.381	0.847
**Age, years**							
<18	27 (13.2)	19 (11.0)	17 (11.1)	15 (10.2)	16 (10.7)	0.997	0.910
18–59	154 (75.5)	133 (76.9)	115 (75.2)	111 (75.5)	112 (75.2)	0.185	0.996
≥60	23 (11.3)	21 (12.1)	21 (13.7)	21 (14.3)	21 (14.1)	1.096	0.895
**Type of BD**							
Mania	55 (27.0)	47 (27.2)	46 (30.1)	45 (30.6)	46 (30.9)	1.218	0.875
Major depression	108 (52.9)	96 (55.5)	81 (52.9)	79 (53.7)	77 (51.7)	0.519	0.972
Mixed state	35 (17.2)	26 (15.0)	24 (15.7)	21 (14.3)	24 (16.1)	0.625	0.960
Rapid cycle	3 (1.5)	1 (0.6)	1 (0.7)	1 (0.7)	1 (0.7)	1.265	0.927
Remission	2 (1.0)	2 (1.2)	1 (0.7)	1 (0.7)	1 (0.7)	0.775	1.000
Other	1 (0.4)	1 (0.6)	0 (0.0)	0 (0.0)	0 (0.0)	2.507	1.000
**Educational background**							
Primary school and below	12 (5.9)	9 (5.2)	9 (5.9)	9 (6.1)	9 (6.0)	0.161	0.997
Junior high school	81 (39.7)	68 (39.3)	57 (37.3)	56 (38.1)	57 (38.3)	0.307	0.989
Senior high school	45 (22.1)	38 (22.0)	38 (24.8)	35 (23.8)	35 (23.5)	0.563	0.967
Junior college or bachelor	62 (30.4)	54 (31.2)	46 (30.1)	44 (29.9)	44 (29.5)	0.124	0.998
Postgraduate or above	4 (1.9)	4 (2.3)	3 (2.0)	3 (2.0)	4 (2.7)	0.493	0.992
**Psychiatric hospitalization, days**							
≤14	61 (29.9)	47 (27.2)	38 (24.8)	38 (25.9)	39 (26.2)	1.388	0.846
15–28	72 (35.3)	62 (35.8)	57 (37.3)	55 (37.4)	57 (38.3)	0.437	0.979
29–56	63 (30.9)	56 (32.4)	50 (32.7)	46 (31.3)	45 (30.2)	0.315	0.989
≥57	8 (3.9)	8 (4.6)	8 (5.2)	8 (5.4)	8 (5.4)	0.647	0.958

Table 2 presents the four medication classes received by patients for each 3-month assessment period. After 12 months, 73.0% of the patients still took medications, and 27.0% of the patients stopped taking medications. Three and 6 months post-discharge were important times at which medication use was discontinued. Mood stabilizers (72–90%) and antipsychotics (77–95%) were still the most important drugs in patients with BD. Antidepressants (34–40%) and benzodiazepines (20–34%) were the other frequently used drug classes. There was no significant difference in antidepressants used during acute episodes and maintenance periods (*P* > 0.05). There were, however, significant differences in mood stabilizers, antipsychotics, and benzodiazepines used (*P* < 0.05).

**TABLE 2:** Numbers of different medication classes used in the treatment of patients with bipolar disorder.

Medication class	Acute episode	Maintenance period				χ^2^	*P*
	Baseline (*n* = 204)	3 months (*n* = 173)	6 months (*n* = 153)	9 months (*n* = 147)	12 months (*n* = 149)		
Mood stabilizers	183 (89.7)	124 (71.7)	112 (73.2)	106 (72.1)	109 (73.2)	25.622	0.000
Antipsychotics	193 (94.6)	142 (82.1)	123 (80.4)	113 (76.9)	117 (78.5)	26.669	0.000
Antidepressants	77 (37.7)	69 (39.9)	57 (37.3)	51 (34.7)	50 (33.6)	1.754	0.781
Benzodiazepines	70 (34.3)	41 (23.7)	34 (22.2)	30 (20.4)	37 (24.8)	11.482	0.022

Table 3 shows the most common drugs used and their daily mean dosage, displayed separately for each 3-months period. For mood stabilizers, 40–56% of BD patients were prescribed lithium, and 33–52% of patients were prescribed valproate. For antipsychotics, 54–65% of BD patients were prescribed quetiapine, and 12–20% of the patients were prescribed olanzapine. Sertraline (6–9%) and fluoxetine (5–9%) were the antidepressants that were most frequently used. Lorazepam (10–18%) was the most commonly used benzodiazepine. There were no changes in the prescription rate of the most common drugs during the 12-month study period, but the prescription rates for lithium, valproate, and oxazepam decreased. Among the major drugs, the mean doses of lithium, valproate, quetiapine, sertraline, and lorazepam were 738.3, 747.6, 233.0, 97.4, and 0.9 mg, respectively. There were no significant changes in the daily mean dosage among the BD patients over 12 months.

**TABLE 3:** The most commonly used drugs and their dosages in the treatment of patients with bipolar disorder, expressed as *n* (%) and mean (SD).

Medication	Acute episode		Maintenance period									
	Baseline (*n* = 204)		3 months (*n* = 173)		6 months (*n* = 153)		9 months (*n* = 147)		12 months (*n* = 149)		χ^2^*/H*	*P*
	*N* (%)	Dose (mg)	*N* (%)	Dose (mg)	*N* (%)	Dose (mg)	*N* (%)	Dose (mg)	*N* (%)	Dose (mg)		
**Mood stabilizers**												
Lithium	115 (56.4)	738.3 ± 242.6	82 (47.4)	709.8 ± 242.7	71 (46.4)	684.5 ± 233.4	64 (43.5)	670.3 ± 231.4	60 (40.3)	696.7 ± 234.3	10.565/5.464	0.032/0.243
Valproate	106 (52.0)	747.6 ± 255.1	59 (34.1)	745.8 ± 252.1	51 (33.3)	670.6 ± 239.0	55 (37.4)	707.3 ± 274.4	53 (35.6)	697.2 ± 258.2	19.048/4.13	0.001/0.389
Lamotrigine	9 (4.4)	111.1 ± 60.1	9 (5.2)	111.1 ± 60.1	9 (5.9)	134.7 ± 72.3	7 (4.8)	164.3 ± 47.6	10 (6.7)	107.5 ± 60.2	1.097/4.836	0.895/0.304
**Antipsychotics**												
Quetiapine	132 (64.7)	233.0 ± 152.3	101 (58.4)	232.4 ± 144.2	90 (58.8)	224.7 ± 139.5	80 (54.4)	229.7 ± 138.0	85 (57.0)	232.4 ± 148.6	4.123/0.121	0.390/0.998
Olanzapine	41 (20.1)	8.9 ± 5.3	27 (15.6)	8.2 ± 4.4	19 (12.4)	8.0 ± 4.2	19 (12.9)	6.3 ± 4.0	19 (12.8)	7.4 ± 4.5	6.040/5.552	0.196/0.235
Aripiprazole	18 (8.8)	11.3 ± 6.0	11 (6.4)	10.7 ± 5.5	12 (7.8)	11.9 ± 5.6	11 (7.5)	11.6 ± 4.8	12 (8.1)	10.8 ± 5.3	0.833/0.552	0.934/0.968
Risperidone	3 (1.5)	3.3 ± 1.2	3 (1.7)	3.3 ± 2.1	4 (2.6)	2.6 ± 2.2	6 (4.1)	2.5 ± 1.4	4 (2.7)	3.0 ± 1.4	2.859/1.188	0.581/0.880
Clozapine	5 (2.5)	95.0 ± 90.8	4 (2.3)	150.0 ± 108.0	5 (3.3)	125.0 ± 109.0	7 (4.8)	125.0 ± 99.0	4 (2.7)	150.0 ± 108.0	2.046/1.660	0.740/0.798
**Antidepressants**												
Sertraline	19 (9.3)	97.4 ± 61.2	16 (9.2)	101.6 ± 64.9	14 (9.2)	100.0 ± 67.2	12 (8.2)	93.8 ± 72.4	9 (6.0)	105.6 ± 63.5	1.566/0.626	0.815/0.96
Fluoxetine	16 (7.8)	28.8 ± 10.2	15 (8.7)	27.3 ± 13.3	11 (7.2)	29.1 ± 13.8	8 (5.4)	25.0 ± 9.3	8 (5.4)	27.5 ± 14.9	2.123/0.975	0.713/0.914
Escitalopram	10 (4.9)	14.0 ± 4.6	8 (4.6)	12.5 ± 3.8	4 (2.6)	11.3 ± 2.5	4 (2.7)	11.3 ± 2.5	3 (2.0)	11.7 ± 2.9	3.418/2.003	0.490/0.735
Venlafaxine	7 (3.4)	150.0 ± 0.0	9 (5.2)	105.6 ± 42.9	8 (5.2)	131.3 ± 53.0	9 (6.1)	105.6 ± 72.4	7 (4.7)	132.1 ± 64.1	1.509/5.903	0.825/0.207
Bupropion	6 (2.9)	250.0 ± 77.5	10 (5.8)	187.5 ± 81.0	7 (4.6)	192.9 ± 73.2	6 (4.1)	225.0 ± 82.2	4 (2.7)	187.5 ± 75.0	2.833/3.334	0.586/0.504
Duloxetine	8 (3.9)	82.5 ± 21.2	6 (3.5)	55.0 ± 35.1	6 (3.9)	55.0 ± 35.1	7 (4.8)	47.1 ± 23.6	6 (4.0)	50.0 ± 15.5	0.356/8.706	0.986/0.069
Paroxetine	5 (2.5)	26.0 ± 13.4	3 (1.7)	40.0 ± 0.0	3 (2.0)	30.0 ± 10.0	3 (2.0)	26.7 ± 11.5	7 (4.7)	23.6 ± 7.5	3.615/5.376	0.461/0.251
**Benzodiazepines**												
Lorazepam	36 (17.6)	0.9 ± 0.5	21 (12.1)	0.9 ± 0.4	15 (9.8)	0.9 ± 0.4	17 (11.6)	0.9 ± 0.4	15 (10.1)	0.9 ± 0.5	6.854/0.477	0.144/0.976
Oxazepam	11 (5.4)	24.5 ± 19.0	2 (1.2)	30.0 ± 0.0	1 (0.7)	7.5 ± 0.0	1 (0.7)	30.0 ± 0.0	1 (0.7)	45.0 ± 0.0	12.308/5.329	0.006/0.255
Clonazepam	13 (6.4)	1.3 ± 0.6	9 (5.2)	1.6 ± 0.5	11 (7.2)	2.0 ± 0.8	5 (3.4)	1.8 ± 0.4	8 (5.4)	2.1 ± 0.8	2.383/9.218	0.666/0.056
Zopiclone	7 (3.4)	7.5 ± 0.0	5 (2.9)	7.5 ± 0.0	4 (2.6)	7.5 ± 0.0	5 (3.4)	6.8 ± 1.7	7 (4.7)	7.0 ± 1.4	1.213/2.908	0.892/0.573
Zolpidem	3 (1.5)	10.0 ± 0.0	4 (2.3)	12.5 ± 5.0	5 (3.3)	11.0 ± 5.5	4 (2.7)	10.0 ± 0.0	7 (4.7)	9.3 ± 1.9	3.541/2.161	0.468/0.706

χ^2^: Difference at baseline, 3, 6, 9, and 12 months. H: Difference in dose at baseline, 3, 6, 9, and 12 months. *P*: *P*-value of the number used drugs and their dosages in the treatment of patients. Only one of the classes of drugs taken by 4% or more of the patients was included.

Considering drug combinations, there were 15 unique combinations by medication class (Table 4). The most frequently taken was MS plus AP co-treatment, accounting for approximately 36–44% of all patients. Twelve months after discharge, the use of MS and AP alone gradually increased. The use of MS + AP + BZD and MS + AP + AD + BZD decreased gradually over the same period, and the use of AD + BZD changed significantly, but not linearly.

**TABLE 4:** Drug classes and combinations used in treating patients with bipolar disorder, expressed as *n* (%).

	Acute episode	Maintenance period					
Combinations by class	Baseline (*n* = 204)	3 months (*n* = 173)	6 months (*n* = 153)	9 months (*n* = 147)	12 months (*n* = 149)	χ^2^	*P*
MS	3 (1.5)	8 (4.6)	10 (6.5)	14 (9.5)	15 (10.1)	15.552	0.004
MS + AP	90 (44.1)	62 (35.8)	60 (39.2)	55 (37.4)	53 (35.6)	3.864	0.426
MS + AD	2 (1.0)	8 (4.6)	4 (2.6)	5 (3.4)	1 (0.7)	7.580	0.091
MS + BZD	0 (0)	2 (1.2)	2 (1.3)	2 (1.4)	3 (2.0)	4.299	0.322
MS + AP + AD	28 (13.7)	18 (10.4)	15 (9.8)	18 (12.2)	18 (12.1)	1.680	0.797
MS + AP + BZD	29 (14.2)	12 (6.9)	9 (5.9)	7 (4.8)	8 (5.4)	15.616	0.003
MS + AD + BZD	5 (2.5)	1 (0.6)	3 (2.0)	0 (0)	3 (2.0)	5.144	0.233
MS + AP + AD + BZD	26 (12.7)	13 (7.5)	8 (5.2)	5 (3.4)	8 (5.4)	14.105	0.007
AP	4 (2.0)	16 (9.2)	10 (6.5)	10 (6.8)	14 (9.4)	13.066	0.010
AP + AD	6 (2.9)	14 (19.2)	14 (9.2)	9 (6.1)	7 (4.7)	7.802	0.099
AP + BZD	0 (0)	3 (1.7)	3 (2.0)	5 (3.4)	3 (2.0)	7.255	0.087
AP + AD + BZD	10 (4.9)	5 (2.9)	3 (2.0)	4 (2.7)	6 (4.0)	2.692	0.619
AD	1 (0.5)	5 (2.9)	6 (3.9)	6 (4.1)	4 (2.7)	6.902	0.126
AD + BZD	0 (0)	5 (2.9)	5 (3.3)	5 (3.4)	3 (2.0)	8.865	0.046
BZD	0 (0)	1 (0.6)	1 (0.7)	2 (1.4)	3 (2.0)	4.588	0.225

AD, antidepressant; AP, antipsychotic; MS, mood stabilizer; BZD, benzodiazepine.

As shown in Table 5, 35–48% of the patients were treated with two drugs and 24–36% received three drugs. While only 2.6% of the patients received one drug at baseline, this increased to 22.8% at the 12th month. The overall average number of drugs used was approximately 2.5.

**TABLE 5:** Number of drugs prescribed to patients being treated for bipolar disorder, expressed as *n* (%).

	Acute episode	Maintenance period					
Number of drugs	Baseline (*n* = 204)	3 months (*n* = 173)	6 months (*n* = 153)	9 months (*n* = 147)	12 months (*n* = 149)	χ^2^	*P*
1	6 (2.9)	25 (14.5)	20 (13.1)	26 (17.7)	34 (22.8)	33.047	0.000
2	71 (34.8)	76 (43.9)	74 (48.4)	69 (46.9)	55 (36.9)	10.199	0.037
3	74 (36.3)	51 (29.5)	37 (24.2)	35 (23.8)	39 (26.2)	9.498	0.050
4	46 (22.5)	11 (6.4)	12 (7.8)	10 (6.8)	14 (9.4)	35.410	0.000
≥ 5	7 (3.4)	10 (5.8)	10 (6.5)	7 (4.8)	7 (4.7)	2.097	0.718
Mean number (SD)	2.9 ± 0.9	2.5 ± 1.0	2.5 ± 1.0	2.3 ± 1.0	2.4 ± 1.1	45.168	0.000

## Discussion

This study found that the first 6 months after the initiation of treatment are very important to medication adherence. Poor medication adherence is a common problem among patients with BD, causing disability and suffering as well as widespread financial costs. Psychotropic adherence was evaluated with medication possession ratio in patients with BD, and about 50% of patients were non-adherence (27, 28). The barriers to adherence are numerous and span multiple levels, including factors related to the pathology of BD, as well as factors specific to the individual’s genetic, psychological, and social environment. Treatment Settings, the health care system, and broader health policies may all influence medication adherence in patients with BD (29). According to medical records, we found that a quarter of inpatients with BD had poor medication adherence. We were surprised to find that this kind of behavior was generally observed within 6 months after treatment. If a patient can adhere to a medication regimen for 6 months, then this adherence could be maintained.

The study also found that mood stabilizers and antipsychotics remained the primary treatment for BD, with 90% of patients using mood stabilizers during an acute episode and approximately 72% of patients using mood stabilizers during the maintenance period. Ninety-five percent of patients used antipsychotics during acute episodes, and approximately 80% used antipsychotics in the maintenance period. Antipsychotics were prescribed at a higher rate than mood stabilizers in both the acute episodes and maintenance periods and thus played the most important role in the treatment of BD. There were many differences in drug use in BD patients. Some previous studies have suggested that mood stabilizers are the primary treatment for BD (30, 31). Meta-analysis data provide some evidence that antipsychotics might be superior to lithium or divalproex in reducing the time required for manic symptoms (32). We clearly observe that treatment is changing over time and that antipsychotics are on the rise in the treatment of BD.

Despite the widespread use of antidepressants in BD, there is controversy surrounding the inclusion of antidepressant medications in management of the disorder (33). The study found that more than 37% of BD patients used antidepressants, and there was no significant difference between the acute episodes and maintenance periods. For the treatment of depressive symptoms, clinicians prefer antidepressants to mood stabilizers that have an anti-depression effect. Although there is a risk to antidepressant use in BD, the improvement in depressive symptoms achieved is incomparable to that of other drugs. In Japan, about 40% of patients with BD are treated with antidepressants. Antidepressants are commonly used in combination with mood stabilizers, antipsychotics, or both. Those taking antidepressants took fewer mood stabilizers, more anti-anxiety medications and more hypnotics than those not taking antidepressants (34).

Although benzodiazepines are not used to treat BD, many people with BD have anxiety and sleep symptoms, so they are often used to treat these secondary symptoms. Although impairment in memory and processing speed was found in patients with BD, benzodiazepine users showed additional neurocognitive impairment in executive function, regardless of whether they received benzodiazepine treatment. These findings support limiting the use of benzodiazepines in patients with BD (35). Our study found that the acute use rate was 34% and the maintenance use rate was 23%. The use during maintenance periods was obviously lower than that during acute episodes. The problem of benzodiazepine addiction is still worthy of attention (36).

In our study, the most commonly used mood stabilizers were lithium and valproate. The most commonly used antipsychotic was quetiapine. Antidepressant use was more dispersed, with sertraline relatively more frequently used, at approximately 8%. The most commonly used benzodiazepine was lorazepam, at approximately 12%. The significant differences between acute episodes and maintenance periods were observed with lithium, valproate, and oxazepam. The proportion of oxazepam used was small, and the influencing factors were not clear. The usage of lithium and valproate during maintenance periods was significantly lower than that in acute episodes. Some previous studies in recent years have shown a decrease in lithium use (37). We found that not only lithium but also valproate showed a decrease. In terms of drug dosage, we did not find significant changes between the acute episodes and maintenance periods. Among the major drugs, the mean doses of lithium, valproate, quetiapine, sertraline, and lorazepam were 738.3, 747.6, 233.0, 97.4, and 0.9 mg, respectively. This can provide a reference for psychiatrists. For antipsychotics used in BD patients, there is a key point we must know. A previous study suggested that aripiprazole was associated with a longer time to hospitalization than ziprasidone, olanzapine, quetiapine, or risperidone in BD patients (38).

There is an increased risk of diabetes mellitus associated with antipsychotic and psychotropic polypharmacy use in BD (39). In our study, drug combinations were still very common in BD, with MS + AP being the predominant drug class combinations, with approximately 36–44% of patients receiving this treatment and no significant changes in acute episodes and maintenance periods. In terms of drug class use, MS, MS + AP + BZD, MS + AP + AD + BZD, AP, and AD + BZD had significant changes in the acute episodes and maintenance periods. MS and AP increased significantly, MS increased from 2 to 10%, and AP increased from 2 to 9%. MS + AP + BZD and MS + AP + AD + BZD decreased significantly, MS + AP + BZD decreased from 14 to 5%, and MS + AP + AD + BZD decreased from 13 to 3%. AD + BZD showed no linear change. From our study, we can see that the treatment during acute episodes and maintenance periods changes, and relatively simple drug use in the maintenance period is more common. The selection, sequence, and combination of anti-manic agents must be tailored to each patient and informed by their illness presentation, comorbidities, previous history, drug costs, treatment preferences, and the availability of safety monitoring (40).

At present, drug treatment is still the main clinical for BD. In order to achieve rapid and effective outcomes, clinicians often use polypharmacy for BD patients (41). Our study found that the average number of medications used by BD patients was 2.5, with more medication used during acute episodes than during maintenance periods. There were 24–36% of patients who used three drugs and 35–48% of patients who used two drugs. The proportion of patients using one drug showed a significant upward trend, and the proportion of those using four drugs showed a downward trend. Recently, there has been a significant increase in polypharmacy. While some of these combinations have been supported by clinical trials, the efficacy of many combinations has not been proven. These trends increase patients’ risk of drug-drug interactions with uncertain benefits in terms of quality of care and clinical outcomes (42).

There were some limitations to this study. First, the sample size of this study was small, and the data were from only one hospital. This affects the credibility of the findings to some extent, and a larger sample size and the inclusion of patients from more hospitals are needed to validate the present result. Sub-analysis such as who were received 2–3 medications and those treated with more than 3 medications would be quite interesting. Second, BD is a complex group of disorders that includes bipolar depression, bipolar mania, mixed state, and rapid cycle, as well as cases in remission and those not classified. Different types may have different treatment drugs. A study of each type is needed. Third, we followed patients with BD for 1 year. We were still not studying patients for a long enough duration, and drug treatment for BD will require much longer observational studies. Finally, there are many different factors that may influence drug choice, such as physician prescribing habits, drug costs, regulatory impact, insurance plan prescribing, drug marketing, and patient preferences, which were not considered in this study. However, from this study, we know about the characteristics of psychotropic drug use and medication adherence of patients with BD at acute and maintenance episodes. These provide reference for psychiatrists in clinical medication. In this way, patients with BD may receive more effective treatment and reduce the burden on patients and society.

## Data availability statement

The original contributions presented in this study are included in this article/supplementary material, further inquiries can be directed to the corresponding author.

## Ethics statement

The studies involving human participants were reviewed and approved by the Ningbo Kangning Hospital Ethics Committee. The Ethics Committee waived the requirement of written informed consent for participation.

## Author contributions

PJ wrote the manuscript, conceived, designed, performed the experiments, and analyzed and interpreted the data. JS, CZ, and XM performed the experiments and contributed to reagents, materials, and analysis tools or data. XZ conceived and designed the experiments, contributed to reagents, materials, and analysis tools or data, and wrote the manuscript. All authors contributed to the article and approved the submitted version.
